# The Basolateral Amygdala to Ventral Hippocampus Circuit Controls Anxiety-Like Behaviors Induced by Morphine Withdrawal

**DOI:** 10.3389/fncel.2022.894886

**Published:** 2022-06-02

**Authors:** Cuola Deji, Peng Yan, Yuanyuan Ji, Xinyue Yan, Yue Feng, Jincen Liu, Yige Liu, Shuguang Wei, Yongsheng Zhu, Jianghua Lai

**Affiliations:** College of Forensic Science, Xi'an Jiaotong University, Xi'an, China

**Keywords:** morphine, anxiety, basolateral amygdala (BLA), ventral hippocampus (vHip), kappa opioid receptor (KOR)

## Abstract

Anxiety is one of the most common comorbid conditions reported in people with opioid dependence. The basolateral amygdala (BLA) and ventral hippocampus (vHip) are critical brain regions for fear and anxiety. The kappa opioid receptor (KOR) is present in the mesolimbic regions involved in emotions and addiction. However, the precise circuits and molecular basis underlying anxiety associated with chronic opioid use are poorly understood. Using a mouse model, we demonstrated that anxiety-like behaviors appeared in the first 2 weeks after morphine withdrawal. Furthermore, the BLA and vHip were activated in mice experiencing anxiety after morphine withdrawal (Mor-A). KORs in the BLA to vHip projections were significantly increased in the Mor-A group. Optogenetic/chemogenetic inhibition of BLA inputs ameliorated anxiety-like behaviors and facilitated conditioned place preference (CPP) extinction in Mor-A mice. Knockdown of the BLA to vHip circuit KOR alleviated the anxiety-like behaviors but did not affect CPP extinction or reinstatement. Furthermore, combined treatment of inhibition of the BLA to vHip circuit and KOR antagonists mitigated anxiety-like behaviors and prevented stress-induced CPP reinstatement after morphine withdrawal. These results revealed a previously unknown circuit associated with the emotional component of opioid withdrawal and indicated that restoration of synaptic deficits with KOR antagonists might be effective in the treatment of anxiety associated with morphine withdrawal.

## Introduction

Opioid drugs are potent analgesics, but they also are exceedingly addictive (powerful euphoria). Opioid abuse relapse occurs with a frequency of more than 85.6% (Vuong et al., 2021). Addiction to opioids depends not only on their positive reinforcing effects but also on avoiding the negative, aversive consequences associated with withdrawal. Early withdrawal symptoms in opioid abusers include diarrhea, yawning, dysphoria, irritability, loss of appetite, severe abdominal pain, and nausea that emerge after drug abstinence (Heishman et al., 1989; Spanagel and Weiss, 1999). Furthermore, numerous psychological symptoms, including anxiety and major depression, gradually increase with the intensity of the drug craving as the withdrawal time lengthens (Goeldner et al., 2011; Radke and Gewirtz, 2012). Notably, heroin-addicted persons with anxiety have higher relapse rates and poor long-term treatment outcomes than heroin-addicted persons who do not have anxiety (Butelman et al., 2012; McKendrick et al., 2020).

The basolateral amygdala (BLA) is a primary site that orchestrates reward-related and emotional processes. Thus, BLA dysfunction is thought to be directly involved in anxiety-like responses and addictive behaviors (Sharp, 2017; Daviu et al., 2019). The BLA is necessary to promote responses to natural rewards, respond to second-order drug-conditioned cues, express stress-enhanced reacquisition of drug intake, and reinstate cue-dependent drug seeking (Sharp, 2017). The BLA mediates fear learning, and the expression of fear as a conditioned response also has been implicated in the genesis and perhaps maintenance of anxiety-like behaviors (Sah, 2017; Sun et al., 2020). The hippocampus has been profoundly implicated in forming addiction-related memories and drug reward experiences (Nestler, 2001a; Dong et al., 2006). The opioidergic system in the ventral hippocampus (vHip) has been demonstrated to be involved in reward-related memory and anxiety-like behaviors. The vHip is a critical site of action for the anxiolytic properties of morphine (Zarrindast et al., 2008; Alvandi et al., 2017). Furthermore, the vHip is a distal BLA projection target implicated in anxiety-related behaviors (McHugh et al., 2004). Previous studies have concluded that the vHip, and not the dorsal hippocampus, is required to express anxiety-related behaviors in the elevated plus maze (EPM) and open field test (OFT) (Bertoglio et al., 2006). The EPM and OFT are the principal behavior tests used to assess anxiety-like behavior in rodents (Carola et al., 2002). However, the functional contribution of BLA inputs to the vHip has not been directly investigated during the withdrawal period after repeated drug administration.

Mu, kappa, and delta are the primary opioid receptor subtypes in brain circuits that share common analgesic effects. This is consistent with the concept that kappa opioid receptor (KOR) activation in animals and humans produces negative affective states and drug-seeking behavioral responses (McLaughlin et al., 2003; Land et al., 2008; Bruijnzeel, 2009; Carroll and Carlezon, 2013). KOR activation exerts anti-reward effects throughout the process of addiction and has the opposite effect of mu opioid receptor (MOR) activation (reward). As addiction develops, intensified stress enhances KOR functions contributing to a dysphoric mood during withdrawal and leading to relapse (Wang, 2019). In humans, selective KOR agonists produce negative mood states, including dysphoria and anxiety (Pfeiffer et al., 1986). Microinfusion of KOR antagonists into the BLA in rodents reduces conditioned fear responses and anxiolytic-like effects in the EPM (Knoll et al., 2011). KOR antagonism prevents morphine stress-induced reinstatement of extinguished and cocaine-conditioned place preference (CPP) (Ross et al., 2012; Brice-Tutt et al., 2020). However, the mechanisms underlying KOR-dependent behaviors have not been clarified, especially at the level of distinct neural circuits and animal models.

We hypothesized that interactions of the BLA to vHip inputs with KORs might be linked to anxiety-like behaviors after morphine withdrawal. We examined anxiety-like behaviors and stress-induced CPP reinstatement during morphine withdrawal in mice. We explored the function of the distal projections from the BLA to the vHip and KORs expression within the projections. We identified a functional role for the BLA to vHip pathway that interacted with KORs in modulating anxiety-like behaviors after morphine withdrawal.

## Materials and Methods

### Animals

Adult male C57BL/6J mice (8 weeks, 22 ± 2 g) were purchased from the Beijing Vital River Laboratory Animal Technology Co., Ltd. The loxP-flanked KOR transgenic mice (KOR^loxp/loxp^ mice) were bought from The Jackson Laboratory. All animals were housed in groups of three to four per cage and kept on a 12-h light/dark cycle (lights on at 7:00 p.m., off at 7:00 a.m.) at a stable temperature (22 ± 3°C) and humidity (50 ± 5%). Experiments were conducted during the light cycle. All animal procedures were approved by the Institutional Animal Care and Use Committee of Xi'an Jiaotong University.

### Drug Preparation and Administration

The HCl-morphine (The Third Research Institute of The Ministry of Public Security, Shanghai, China) and naloxone (N822820, MACKLIN) were dissolved in saline. Morphine was administered at an escalating dose (from 10 to 50 mg/kg) *via* intraperitoneal (i.p.) injection two times daily for 6 consecutive days. Control animals received two times daily i.p. injections of saline for 6 consecutive days accordingly. Naloxone was injected subcutaneously (s.c.) into the morphine group mice and the saline group mice at a dose of 2 mg/kg. CNO (3 mg/kg, Sigma, C0832) and nor-BNI (10 mg/kg, 113158-34-2, MCE) were dissolved in the 0.5% DMSO. The DMSO was diluted to 0.5% with saline. KOR antagonist nor-BNI is a long-lasting antagonist (Horan et al., 1992), is delayed in its onset of action, and produces peak effects after 24 h (Endoh et al., 1992; Butelman et al., 1993; Metcalf and Coop, 2005). In addition, nor-BNI is most selective for KORs 24 h after administration (Endoh et al., 1992; Valdez and Harshberger, 2012). Thus, we injected 10 mg/kg nor-BNI intraperitoneally 24 h before the behavior test.

### Measurement of Withdrawal Signs

Naloxone, μ opioid receptor antagonist, was used to induce the somatic symptoms (Boyle et al., 2009). Mice received naloxone (2 mg/kg, s.c.) before the observation of somatic symptoms and were placed in a white opaque cylinder (32.0 cm height × 10.0 cm diameter); signs of withdrawal syndrome were monitored for 20 min. For the evaluation of behavioral signs of withdrawal, nine parameters were evaluated (The number of wet dog shakes, front paw tremors, scratches, jumping, and sniffing was counted. Body tremor, ptosis, teeth chattering, and piloerection were scored 1 for appearance or 0 for non-appearance within 5 min bins.). A global withdrawal score was calculated for each animal by giving each somatic sign a relative weight: 0.5 for each episode of wet dog shake, paw tremor, scratching, sniffing, and jumping; and 1 for the presence of body tremor, ptosis, mastication, and piloerection during each 5-min observation period. Each mouse was scored individually. Data were analyzed in a double-blinded manner. The results were assessed such that the higher the score, the more severe the withdrawal symptoms.

### Conditioned Place Preference

The CPP procedure was performed according to our previous study (Qiao et al., 2021). During the conditioning test, an escalating morphine was administrated to induce the morphine-paired side preference. On the test day, mice were allowed to freely explore the chambers for 15 min without injections. The time spent in each chamber was determined using a video tracking system. During the extinction training, all mice were given saline (i.p., 10 ml/kg) once daily and were immediately confined to the chambers for 45 min. The day after every 2 rounds (4 days) of extinction training, the place preference of the mice was tested for 15 min until the mice exhibited no preference for the morphine-paired side. The mice that showed CPP extinction were subjected to stress-induced reinstatement. On the next day, mice were shuttled from the CPP testing room to an intentionally different adjacent room with the shock apparatus, put in the shock box for 5 min of habituation, and then exposed to 15 min of random shocks (0.8 mA) that lasted 0.5 s each with an intershock interval from 10 to 70 s (mean of 40 s) (Nygard et al., 2016). After the 20-min foot shock stress, the stress-induced reinstatement was tested by allowing the mice to freely explore the CPP chambers for 15 min.

### Elevated Plus Maze Test

The EPM consists of two open arms (33 cm × 6 cm) and two closed arms (33 cm × 6 cm) intersecting at 90 degrees in the form of a plus, with a central area (6 cm × 6 cm). The maze was elevated 50 cm from the floor. Each mouse was placed in the center of the apparatus for a test for 5 min. The number of entries and the time spent in the open arm were recorded by ANY-maze software (Stoelting Company, Wood Dale, IL, USA) as a measure of anxiety. Between each trial, the maze was cleaned with 50% ethanol.

### Open Field Test

Mice were placed into an open-field box (45 × 45 × 30 cm) under dim light (80 lx) for 15 min. The ANY-maze software was used to record the movement trail and analyze the locomotor activity of mice. The total time spent in the central field (30 × 30 cm) was measured as an index of anxiety.

### Surgery and Microinjection

Mice were fixed in a stereotaxic frame (RWD, Shenzhen, China) under isoflurane anesthesia. Two holes were drilled in the skull of each mouse above the intended site of injection (BLA: AP – 1.4 mm, ML ± 3.4 mm, DV – 4.7 mm; vHip: AP – 3.1 mm, ML ± 3.3 mm, DV – 4.2 mm); 150–200 nl of the virus was delivered by 40 nl/min at each intended site through a Hamilton microsyringe with a microinjection pump (RWD, Shenzhen, China). After each injection, the needle was left in place for >10 min to allow for the diffusion of the virus and then slowly withdrawn.

### Fiber Optic Ferrules Implantation and Optical Behaviors Test

For the optical experiment, animals were implanted bilaterally with optical fibers (200 mm core, numerical aperture = 0.37) held in a ceramic ferrule (Fibers, Shanghai, PRC) in vHip (−3.1 AP, ± 3.3 ML, and −3.7 DV), and the implants were secured to the skull with dental cement. All mice were handled for 3 days before behavioral assays for 5 min per day to reduce stress introduced by contact with the experimenter; 1–5 min were allowed for recovery in the home cage from handling. The EPM test consisted of a 9-min session divided into three 3-min epochs: the pre-stimulation light-off epoch, the light-on epoch, and the post-stimulation light-off epoch, in order (off-on-off epochs) (Felix-Ortiz et al., 2013). The OFT consisted of a 15-min session in which there were three 5-min epochs (off-on-off epochs). For optogenetic inhibition of BLA to vHip inputs, we used a constant illumination of yellow light stimulation (5 mw, NewDoon Aurora 220).

### Immunofluorescent Histochemistry

Mor-A mice and saline control mice were used to analyze c-Fos expression. Mor-A mice and saline control mice were tested for anxiety-like behavior using the EPM 2w after morphine withdrawal. These mice were returned directly to their home cages after testing. After 90 min, mice were perfused transcardially with saline, followed by 4% (w/v) paraformaldehyde in 0.1 M phosphate buffer. Brains were post-fixed in 4% paraformaldehyde for 3 h and then transferred to 30% sucrose for 24 h. Twenty micrometer thick floating sections were obtained using a freezing microtome (CM1950, Leica). The sections were rinsed in 0.01 M PBS three times and blocked in 0.01 M PBS containing 10% normal donkey serum and 0.3% (v/v) Triton X-100 for 1 h at room temperature. The blocked sections were then incubated overnight at room temperature with the mouse anti-c-Fos (1:200; ab208942, Abcam) in PBS containing 0.3% (v/v) Triton X-100, 0.25% (w/v) λ-carrageenan, and 5% (v/v) donkey serum (PBS-XCD). Sections were incubated for 5 h at room temperature with Alexa594-conjugated donkey anti-mouse IgG (1:200; A11055, Invitrogen). The sections were observed with a Zen microscope (ZEN 3.2, ZEISS). The c-Fos^+^ cell number per mm^2^ and the percent of c-Fos^+^ + FG^+^/FG^+^ cell number were calculated.

### Fluorescence *in situ* Hybridization (FISH)

Mor-A mice and saline control mice were used for KOR analysis after the behavior test. We synthesized the digoxigenin (DIG)-labeled antisense single-strand RNA probes of KOR (http://mouse.brain-map.org/) with a DIG RNA labeling kit (11277073910, Roche Diagnostic). Target sections were treated with 2% H_2_O_2_ in 0.1 M of DEPC-PB for 10 min. After rinsing with 0.1 M DEPC-PB and reacting in acetylation solution, the sections were pre-hybridized for 1 h at 58°C in hybridization buffer. Then, 1 μg/ml KOR RNA probe was added and hybridized at 58°C for 20 h. After rinsing in wash buffer for 20 min two times at 58°C, the hybridized sections were incubated with 20 μg/ml ribonuclease A for 30 min at 37°C. The sections were incubated overnight with 0.5 μg/ml peroxidase-conjugated anti-digoxigenin sheep antibody (11-207-733-910; Roche Diagnostics). We performed the biotinylated tyramine (BT)-glucose oxidase (GO) amplification method to amplify the KOR hybridization signals. The sections were subsequently treated with 10 μg/ml Fluorescein Avidin D (A2901, Sigma) for 5 h. Then, the sections were observed with a Zen microscope (ZEN 3.2, ZEISS).

### Western Blotting

The brains were removed, and BLA and vHip were carefully dissected. The Western blotting procedure was conducted as described in our previous study (Qiao et al., 2021). The dilutions of primary antibodies were as follows: KOR (1:1,000, ab183825, Abcam) and GAPDH (internal control, 1:2,000, ab8245, Abcam). All species-appropriate horseradish peroxidase-conjugated secondary antibodies were used at a dilution of 1:10,000. The KOR protein expression level was normalized to GAPDH expression and presented as relative quantifications.

### RNA Isolation and q-PCR

The RNA isolation, reverse transcription, and quantitative real-time PCR were carried out as described previously (Qiao et al., 2021). The level of KOR mRNA expression was analyzed by the fold change relative to GAPDH expression. The relative mRNA level was analyzed as the difference from the experimental relative to the control condition. KOR primer sequences were designed by Takara Bio Inc. (Beijing, China) and are described as follows: Oprk: forward (5′-3′): CATTTGGCTCCTGGCATCATC, reverse (5′-3′): AGGAGCATTCAATGACATCCACA; Gapdh: forward (5′-3′): TGTGTCCGTCGTGGATCTGA, reverse (5′-3′): TTGCTGTTGAAGTCGCAGGAG.

### Statistical Analysis

Statistical analyses were conducted using GraphPad Prism 8.0 (GraphPad Software Inc., La Jolla, CA). The data passed the normality and homogeneity of variance test. The behavior and withdrawal syndrome test were analyzed by unpaired Student's *t*-test. Chemogenetics and KOR knockdown experiments were analyzed by two-way ANOVA. Between-group comparisons were done only when there was a statistical interaction (Sidak's *post-hoc* test). Parameters of CPP and optogenetic experiments were analyzed by the repeated measure two-way ANOVA (RM-ANOVA). Multiple comparison was done by Sidak's *post-hoc* test. Others were analyzed by unpaired Student's *t*-test. The results are presented as the mean ± standard error of the mean (SEM) (Supplementary Table 1). Differences were considered significant at *p* < 0.05. Investigators were blinded to the allocation of groups and outcome assessment for all experiments.

## Results

### Mice Experiencing Morphine-Withdrawal Showed Anxiety-Like Behaviors

**Figure 1:**
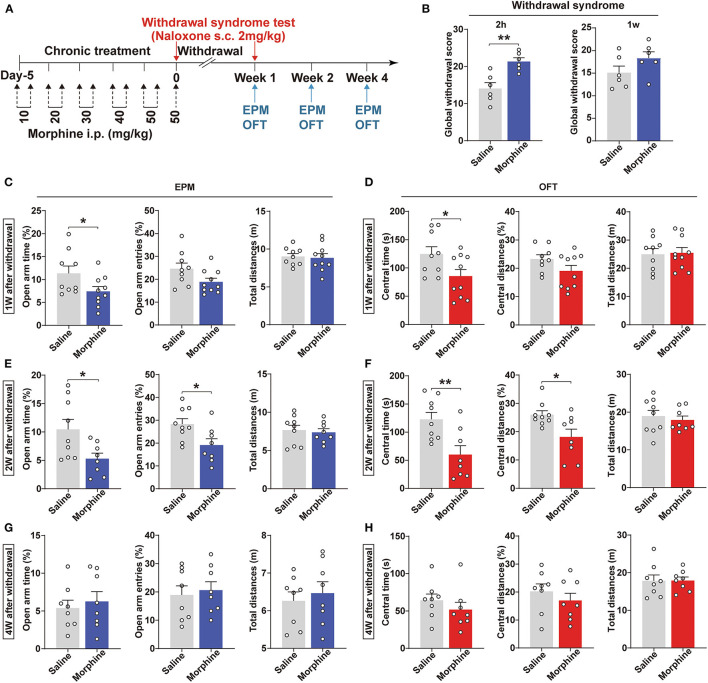
Morphine-withdrawn mice show anxiety-like behaviors. **(A)** Schematics of the experiment. **(B)** The global withdrawal score of morphine was higher than saline after withdrawal 2 h from morphine; 2 mg/kg of naloxone was injected subcutaneously (s.c.) into morphine and saline mice. Anxiety-like behavioral tests using the EPM and OFT at 1 week **(C,D)**, 2 weeks **(E,F)**, and 4 weeks **(G,H)** of withdrawal. Data were expressed as the mean ± SEM. **(B)** *n* = 6/group, **(C–F)** *n* = 8–10/group *P < 0.05, **P < 0.01, compared with saline group.

Population studies have revealed that opioid addicts suffer from anxiety after withdrawal (Williams et al., 2001; Chu et al., 2009). This study utilized a chronic morphine regimen to develop a mouse morphine-withdrawal anxiety model (Figure 1A). Mice were treated with naloxone (2 mg/kg, s.c.) 2 h after the last morphine injection. We observed a robust somatic syndrome in the morphine-withdrawn mice (*p* = 0.0030, Figure 1B), while this syndrome was not present 1 week after morphine withdrawal. Due to the administration of naloxone, these groups were not used to test the withdrawal anxiety-like behavior. Next, we used another group to assess the negative emotional signs of morphine withdrawal. Anxiety-like behaviors were assessed using the EPM and OFT at 1, 2, and 4 weeks after withdrawal. After 1 week of withdrawal, mice spent less time in the open arms of the EPM (*p* = 0.0476, Figure 1C) and the central area of the OFT (*p* = 0.0397, Figure 1D) compared with saline control mice. Intriguingly, anxiety-like behavior was more pronounced after 2 weeks of withdrawal from morphine. After 2 weeks of morphine withdrawal, mice spent significantly less time in the open arms (*p* = 0.0254, Figure 1E) and exhibited decreased numbers of open-arm entries (*p* = 0.0230, Figure 1E). In addition, the differences in the central time and central distances in the OFT were statistically significant (*p* = 0.0062, *p* = 0.0181, Figure 1F). However, after 4 weeks of morphine withdrawal, the mice did not exhibit any anxiety-like behaviors (*p* > 0.05, Figures 1G,H). Also, the total distances were not different between these two groups, indicating that morphine withdrawal did not alter locomotion in the mice. Therefore, mice withdrawn from morphine for 2 weeks that showed anxiety (Mor-A) were used for subsequent experiments.

### BLA to vHip Projections Were Involved in Anxiety-Like Behaviors Induced by Morphine Withdrawal

**Figure 2:**
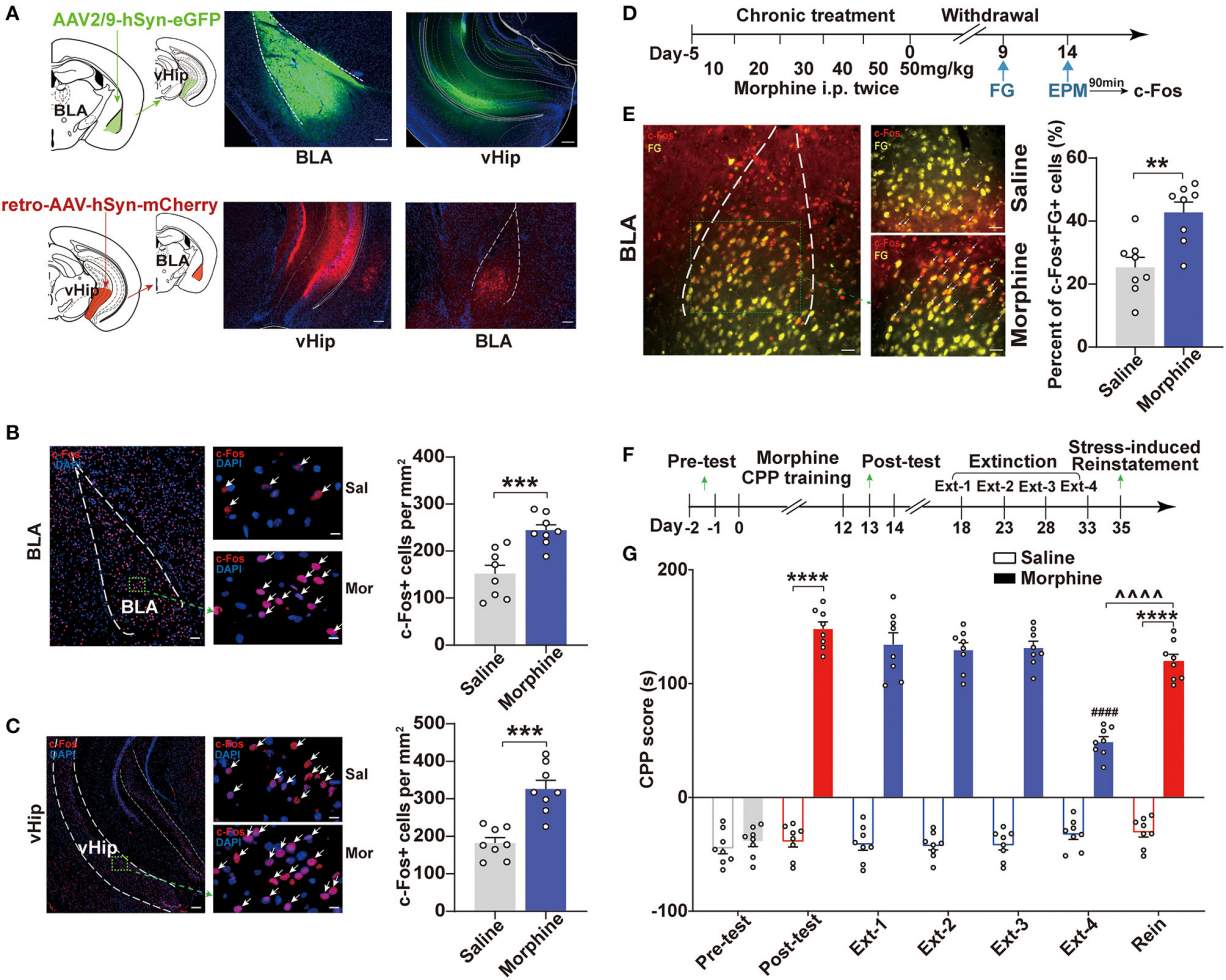
BLA to vHip projections are involved in the anxiety-like behaviors induced by morphine withdrawal. **(A)** Schematic for tracing projections from the BLA to the vHip in wild-type mice. Representative coronal images of the injection site and tracing terminals. (Top) The anterograde virus AAV2/9-hSyn-eGFP was delivered in the BLA. (Bottom) The retrograde virus retro-AAV-hSyn-mCherry was delivered in the vHip. Scale bar: 200 μm. Representative immunofluorescence images of c-Fos (red) in the BLA [**(B)**, left] and vHip [**(C)**, left]. The nuclei were stained with DAPI (blue). Scale bar: 200 μm. Arrows indicate neurons co-labeled with c-Fos. Regions enclosed in a green box are shown at higher magnification in the images to the right. Scale bars: 50 μm. The numbers of c-Fos-positive neurons per mm^2^ in the BLA [**(B)**, right] and vHip [**(C)**, right]. **(D)** Timeline of c-Fos and fluorogold (FG) co-expression in BLA of Mor-A mice. [**(E)**, Left] Representative coronal image of c-Fos (red) and fluorogold (FG, yellow) immunostaining in the BLA. Scale bar: 100 μm. Regions enclosed by a green box are shown at higher magnification in the images to the right. Arrows indicate neurons co-labeled with c-Fos and FG. Scale bars: 50 μm. (Right) The percentage of neurons co-labeled with c-Fos^+^ and FG^+^ relative to FG in the BLA. **(F)** Schematic of the experimental design for the training and testing of morphine-CPP acquisition, extinction, and reinstatement. **(G)** CPP scores in the morphine-CPP expression, extinction, and reinstatement tests. Morphine-induced CPP, extinction at Ext-4 and reinstatement induced by stress. In the CPP results, #: comparison between the Extinction-4 of morphine group and the post-test of morphine group, ^∧^: comparison between the reinstatement of morphine group and the Extinction-4 of morphine group; *: comparison between the 2 indicated groups. BLA, basolateral amygdala. vHip, ventral hippocampus. Data are expressed as the mean ± SEM. **P < 0.01, ***P < 0.001, ****P < 0.0001, ^∧∧∧∧^*P* < 0.0001, ^*####*^*P* < 0.0001. *n* = 6–8/group.

Previous studies suggested that BLA excitability was positively associated with increased anxiety-like responses and addiction (Sharp, 2017; Daviu et al., 2019). One distal BLA projection target implicated in anxiety-related behaviors was the vHip. The BLA provides glutamatergic inputs to the vHip (Felix-Ortiz et al., 2013). We confirmed the projections from the BLA to vHip and investigated whether the BLA and vHip were activated. We used virus-delivered trackers to delineate the circuit to map the connection between BLA and vHip. By injecting the anterograde tracker (AAV2/9-hSyn-eGFP, titer: 1.91 × 10^13^ vg/ml, OBiO) into the BLA, we observed robust expression of mCherry in the vHip (Figure 2A, top). Conversely, a retro adeno-associated virus (retroAAV-hSyn-mCherry, titer: 6.33 × 10^13^ vg/ml, OBiO) was injected into the vHip retrogradely labeled neurons in the BLA (Figure 2A, bottom). These results demonstrated that the BLA shared projections to the vHip. To confirm neuronal activation in both the BLA and vHip in Mor-A mice and the saline control mice, we measured the expression of an immediate-early gene product, c-Fos, which is a surrogate molecular marker of neuronal activity. We stained for c-Fos in the BLA and vHip using immunofluorescence. Robust c-Fos signals were detected in the BLA (*p* = 0.0008, Figure 2B) and vHip (*p* = 0.0001, Figure 2C) of Mor-A mice. To further determine that the BLA to vHip projections were activated in the Mor-A mice, we injected the retrograde tracer fluorogold (FG) into the vHip and performed FG/c-Fos double labeling in the BLA of the Mor-A mice and control mice (Figure 2D). Our results revealed an increased percentage of c-Fos^+^ + FG^+^ cells (calculated as c-Fos^+^FG^+^ double-labeled cells/total FG positive cells) in the BLA of Mor-A mice compared with the saline control mice (*p* = 0.0020, Figure 2E), verifying that the BLA to vHip projections were involved in the process of anxiety-like behaviors following morphine withdrawal.

Individuals with stress or anxiety disorders are particularly vulnerable to opioid addiction (Conway et al., 2006). In this study, the morphine group showed a significant preference for the morphine-paired chamber (*p* < 0.0001, Figures 2F,G). After 4 rounds of extinction (~2 weeks of withdrawal), the morphine-paired preference was significantly diminished in the Mor-A mice (*p* < 0.0001, Figure 2G). However, the morphine-paired preference of the Mor-A mice was restored by foot shock (*p* < 0.0001, Figure 2G). Furthermore, the reinstatement CPP score of the Mor-A mice significantly differed from the Ext-4 CPP score of the Mor-A mice (*p* < 0.0001, Figure 2G). This observation indicated that the preference in the morphine CPP mice was diminished after 4 rounds of extinction training in this study. Taken together, the BLA to vHip projections were involved in the anxiety-like behaviors induced by morphine withdrawal, and the Mor-A mice were prone to stress-induced reinstatement.

### BLA to vHip Projections Regulated Anxiety-Like Behaviors in Morphine-Withdrawn Mice

**Figure 3:**
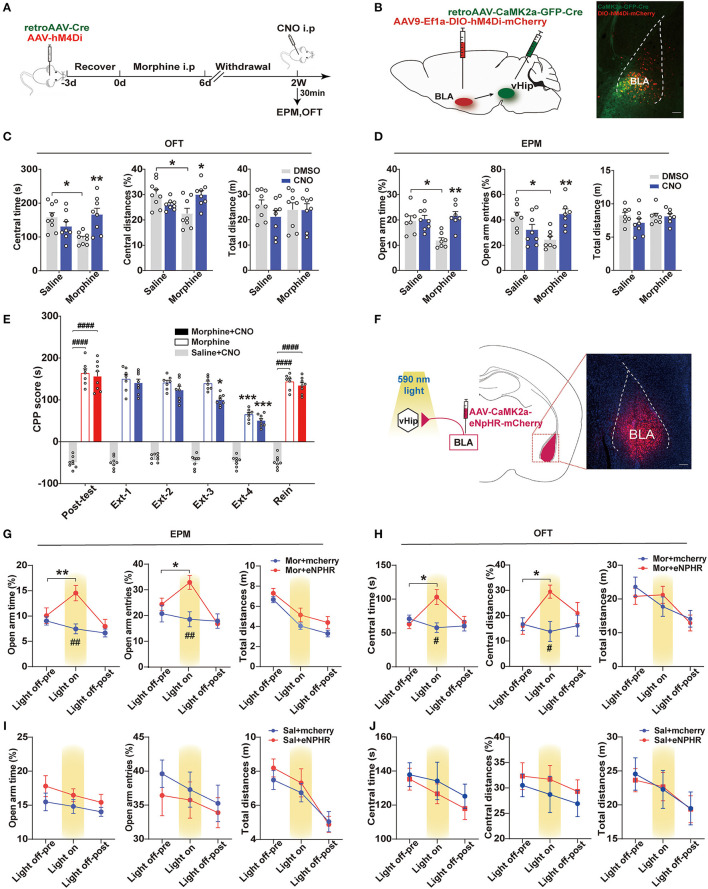
BLA to vHip projections regulate anxiety-like behavior in morphine-withdrawn mice. **(A–E)** Chemogenetic experiment. **(A)** Workflow for the chemogenetic experiment. **(B)** Schematic (left) and representative image (right) of chemogenetic virus injection. Scale bar: 200 μm. **(C)** The Mor-A×CNO mice exhibited significantly increased central time and central distances in the OFT. **(D)** The Mor-A × CNO mice exhibited significantly increased open-arm times and entries in the EPM. **(E)** The hM4Di inhibition accelerated the decreased rate of morphine-paired preference in the Mor-A mice. **(F–H)** Optogenetic experiment. **(F)** Schematic of the virus injection site in the BLA and optical fiber implantation site in the vHip. (Right) Image of a coronal brain slice showing the expression of eNpHR-mCherry in the BLA. Scale bar: 200 μm. **(G)** Increased open-arm entries and time spent during the light-on epoch. **(H)** Increased time spent and distances traveled in the central area of the OFT during the eNpHR illumination epoch. The EPM **(I)** and OFT **(J)** tests of the saline + eNpHR group and the saline + mCherry group. Data are expressed as the mean ± SEM. In the chemogenetic experiment, morphine CNO-treated mice were compared with morphine DMSO-treated mice. In the CPP results, *: comparison between the extinction group and the post-test group, #: comparison between the 2 indicated groups. In the optogenetic results, #: comparison between the Mor+mcherry and the Mor+eNPHR group during the light on epoch. *n* = 8–10/group.*P < 0.05, **P < 0.01, ***P < 0.001, ^#^*P* < 0.05, ^##^*P* < 0.01, ^####^*P* < 0.0001.

Population studies have shown that increased amygdala: hippocampus volume ratios are associated with increased anxiety severity (MacMillan et al., 2003). Animal studies have demonstrated that activation of the BLA-vHip inputs robustly increased anxiety-related behaviors (Felix-Ortiz et al., 2013). To explore whether BLA to vHip projections regulated anxiety-like behaviors following morphine withdrawal, we used a combination of retrograde viral and chemogenetic approaches to silence the activity of neurons involved in the BLA to vHip projections. Specifically, we selectively expressed the inhibitory designer Gi-coupled human muscarinic receptor 4 (hM4Di) in BLA to vHip projections and assessed anxiety-like behaviors in the presence of the selective exogenous ligand clozapine-N-oxide (CNO) that inhibited BLA to vHip projections (Figure 3A). The hM4Di-receptor was expressed exclusively in the glutamatergic projections from the BLA to vHip using a Cre-dependent approach [AAV9-EFIa-DIO-hM4Di (Gi)-mCherry, titer: 5.80 × 10^13^ vg/ml, OBiO; retroAAV-CaMK2-GFP-2A-Cre, titer: 4.36 × 10^12^ vg/ml, OBiO] (Figure 3B). Two weeks later, the mice were intraperitoneally injected with 3 mg/kg CNO or DMSO, and behavioral tests were conducted 30 min later. Two-way ANOVA showed significant main effects of morphine × CNO interactions [OFT: central time: *F*_(1,29)_ = 12.40, *p* = 0.0014, central distances%: *F*_(1,29)_ = 12.66, *p* = 0.0013; EPM: open-arm time%: *F*_(1,25)_ = 7.876, *p* = 0.0096, open arm entries%: *F*_(1,25)_ =16.16, *p* = 0.0005]. Compared with the Mor-A × DMSO mice, *post-hoc* analysis revealed that inhibition of the BLA to vHip projections in morphine-withdrawn mice (Mor-A × CNO) prevented the decreased time (*p* = 0.0085, Figure 3C) and decreased traveled distances (*p* = 0.0224, Figure 3C) in the central zone of the OFT. The decreased open-arm time (*p* = 0.0019, Figure 3D) and decreased entries (*p* = 0.0065, Figure 3D) in the EPM were also prevented. Chemogenetic inhibition of the BLA to vHip projections did not affect locomotor activity in Mor-A mice (*p* > 0.05, Figure 3C). Then, we used RM-ANOVA to analyze whether the inhibition of BLA to vHip inputs affected the morphine CPP process. The Mor-A × CNO mice showed a diminished morphine-paired preference at Ext 3 (*p* = 0.0216, Figure 3E), but the inhibition did not prevent the stress-induced reinstatement (*p* < 0.0001, Figure 3E). These results indicated that the bilateral inhibition of BLA terminals in the vHip ameliorated morphine-withdrawal-induced anxiety-like behaviors and accelerated the decreased rate of morphine-paired preference in morphine CPP.

To further confirm these results, an optogenetic approach inhibiting BLA to vHip projections was used to assess changes in anxiety-like behaviors. AAVs carrying either the inhibitory opsin, inhibitory natronomonas pharaonis halorhodopsin (eNpHR3.0), or mCherry fluorescent protein (red) under the control of the CamKIIα promoter were injected bilaterally into the BLA (AAV-CaMK2-eNpHR-mCherry, titer: 2.63 × 10^12^ vg/ml; AAV-CaMK2-mCherry, titer: 2.79 × 10^12^ vg/ml BrainVTA), and optical fibers were implanted in the vHip of mice (Figure 3F). The Mor-A mice were tested in the EPM for 9 min, with alternating 3-min periods of no illumination, illumination, and no illumination. RM-ANOVA revealed a significant main effect of light × morphine interactions on EPM [open-arm time%: *F*_(2,28)_ = 17.07, *p* < 0.0001, open-arm entries%: *F*_(2,26)_ = 8.749, *p* = 0.0012]. Mor-A mice expressing eNpHR (Mor-A × eNpHR) exhibited decreased open-arm avoidance, as evidenced by more entries (*p* = 0.0253, Figure 3G) and time spent in the open arms (*p* = 0.0077, Figure 3G). The mice were also tested in the OFT for 15 min (5 min off−5 min on−5 min off). RM-ANOVA revealed a significant main effect of light × morphine interactions on OFT [central time: *F*_(2,28)_ = 8.633, *p* = 0.0012, central distances%: *F*_(2,28)_ = 5.491, *p* = 0.0097]. Bilateral inhibition of BLA terminals in the vHip decreased center avoidance in the Mor-A mice with more spent time (*p* = 0.0290, Figure 3H) and increased traveled distances (*p* = 0.0101, Figure 3H) in the central area of the OFT. Locomotor behavior was not affected by illumination in either the mCherry or eNpHR group (*p* > 0.05, Figures 3H–J). These optogenetic results suggested that specific inhibition of this pathway ameliorated anxiety-like behaviors in the Mor-A mice. Collectively, the chemogenetic and optogenetic inhibition results suggested that the BLA to vHip projections were important for anxiety-like behavior induced by morphine withdrawal.

### Specific Knockdown of Kappa Opioid Receptors in the BLA to vHip Projections Improved Anxiety-Like Behaviors in Morphine-Withdrawn Mice

**Figure 4:**
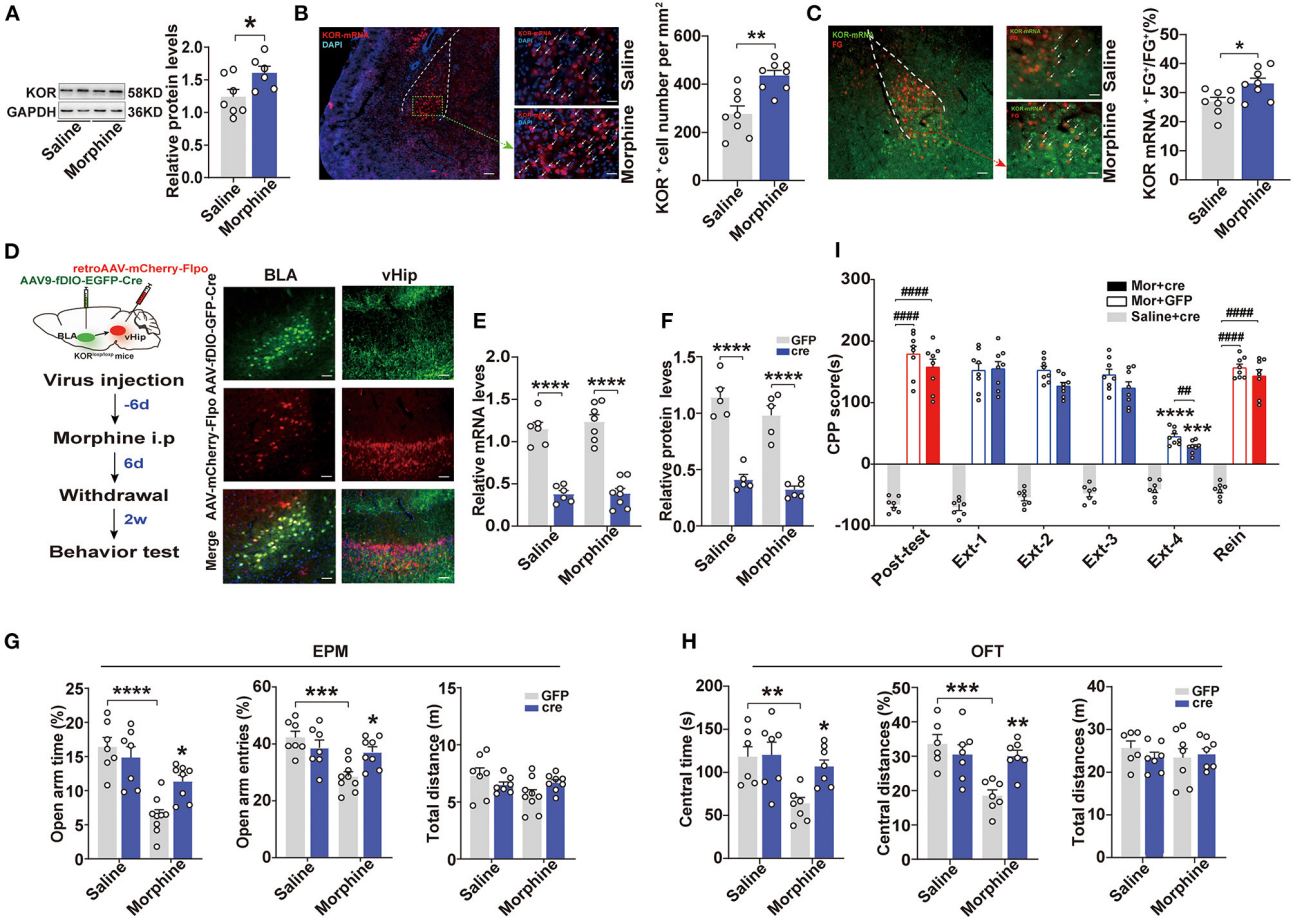
Specific knockdown of the kappa opioid receptor in the BLA to vHip projections ameliorate anxiety-like behaviors in morphine-withdrawn mice. **(A)** Immunoblots and quantification analysis of KOR levels in the BLA. KOR protein expression levels increased in the BLA of the Mor-A mice. **(B)** (Left) Representative images of KOR mRNA (red) in the BLA of the Mor-A mice. The nuclei were stained with DAPI (blue). Scale bar: 200 μm. BLA regions enclosed by a green box are shown at higher magnification in images to the right. Scale bars: 50 μm. (Right) KOR^+^ cell numbers per mm^2^ in the BLA of the Mor-A mice were increased. **(C)** Co-labeled neurons for KOR mRNA and FG in the BLA. Retrograde FG was injected into the vHip. (Left) Representative images of KOR mRNA (green) and fluorogold (FG; red) immunostaining in the BLA. Scale bar: 200 μm. BLA regions enclosed by a red box are shown at higher magnification in the images to the right. Arrows indicate neurons co-labeled with FG and KOR mRNA. Scale bars: 50 μm. (Right) The percentage of neurons co-labeled with KOR mRNA and FG relative to FG in the BLA. **(D)** (Left) Schematic of the KOR knockdown from the BLA to vHip projections in the KOR^loxp/loxp^ mice. (Right) Representative coronal images of virus injection in the BLA and vHip. Scale bar: 100 μm **(E)** KOR mRNA and **(F)** KOR protein expression levels in the BLA of the KOR^loxp/loxp^ mice with Cre-recombinase expression were decreased. **(G)** The Mor-A mice with KOR knockdown exhibited increases in open-arm time and entries in the EPM. **(H)** The Mor-A mice with KOR knockdown exhibited increases in central time and central distances in the OFT. **(I)** Knockdown of KOR in the BLA to vHip projections did not affect the morphine CPP. Data are expressed as the mean ± SEM. The KOR knockdown experiment compared the Mor-A mice expressing Cre-recombinase with Mor-A mice expressing GFP. In the CPP results, *: comparison between the extinction group and the post-test group. #: comparison between the 2 indicated groups. *n* = 6–8/group. *P < 0.05, **P < 0.01, ***P < 0.001, ****P < 0.0001; ^##^*P* < 0.01, ^####^*P* < 0.0001.

Evidence has suggested that KORs in the BLA modulate anxiety-like behaviors (Knoll et al., 2011). Therefore, we assessed the expression levels of KORs in the BLA. Western blot analysis revealed that the Mor-A mice exhibited a higher relative level of KOR in the BLA (*p* = 0.0372, Figure 4A). These results were confirmed by *in situ* hybridization. The KOR mRNA expression levels were significantly higher in the BLA of Mor-A mice (*p* = 0.0012, Figure 4B). Then, we injected fluorogold (FG) into the vHip and performed KOR mRNA *in situ* hybridization in the BLA. We observed increased numbers of FG^+^ and KOR mRNA double-labeled cells in the BLA of the Mor-A mice (*p* = 0.0191, Figure 4C), indicating that the KOR mRNA expression level in BLA to vHip projection neurons was also increased. We further determined whether KORs in the BLA to vHip projections modulated anxiety-like behaviors induced by morphine withdrawal. We used KOR^loxp/loxp^ mice to knock down KORs in the BLA to vHip projections conditionally. Specifically, the KOR^loxp/loxp^ mice were stereotaxically injected with AAV-Ef1a-fDIO-EGFP-2A-Cre (titer: 1.12 × 10^13^ vg/ml, OBiO) into the BLA and retro-AAV-CaMK2-mCherry-Flpo (titer: 4.37 × 10^12^ vg/ml, OBiO) into the vHip for widespread Cre-recombinase expression (Figure 4D). Two-way ANOVA revealed a significant main effect of Cre-recombinase expression on the KOR expression level [mRNA: *F*_(1,23)_ = 0.3128, *p* = 0.5814; protein: *F*_(1,17)_ = 0.3308, *p* = 0.5727]. The *post-hoc* test indicated that the KOR mRNA (*p* < 0.0001, *p* < 0.0001; Figure 4E) and protein expression levels (*p* < 0.0001, *p* < 0.0001; Figure 4F) were dramatically decreased in the KOR^loxp/loxp^ mice expressing Cre-recombinase, confirming that KOR transgene mice were an effective tool for manipulating KOR expression. Two-way ANOVA showed a significant main effect of KOR knockdown × morphine interaction on anxiety-like behavior induced by morphine withdrawal [EPM: open-arm time%: *F*_(1,27)_ = 7.792, *p* = 0.0095, open-arm entries%: *F*_(1,27)_ = 8.367, *p* = 0.0075; OFT: central time: *F*_(1,23)_ = 4.928, *p* = 0.0366; central distances%: *F*_(1,23)_ = 10.26, *p* = 0.0039]. The KOR^loxp/loxp^ Mor-A mice expressing Cre-recombinase spent more time (*p* = 0.0226, Figure 4G) and exhibited more entries (*p* = 0.0323, Figure 4G) into the open arms of the EPM, and spent more time (*p* = 0.0301, Figure 4H) and traveled a greater distance (*p* = 0.0078, Figure 4H) in the central area of the OFT. These results indicated that knocking down KORs in the BLA to vHip projections ameliorated the anxiety-like behaviors but did not affect CPP extinction or stress-induced reinstatement in the Mor-A mice (*p* > 0.05, Figure 4I). Taken together, these results revealed that KORs in the BLA to vHip projections maintained the basal level of anxiety-like behaviors induced by morphine withdrawal.

### Simultaneous Intervention of BLA to vHip Projections and KORs Prevented the Reinstatement of Morphine CPP

**Figure 5:**
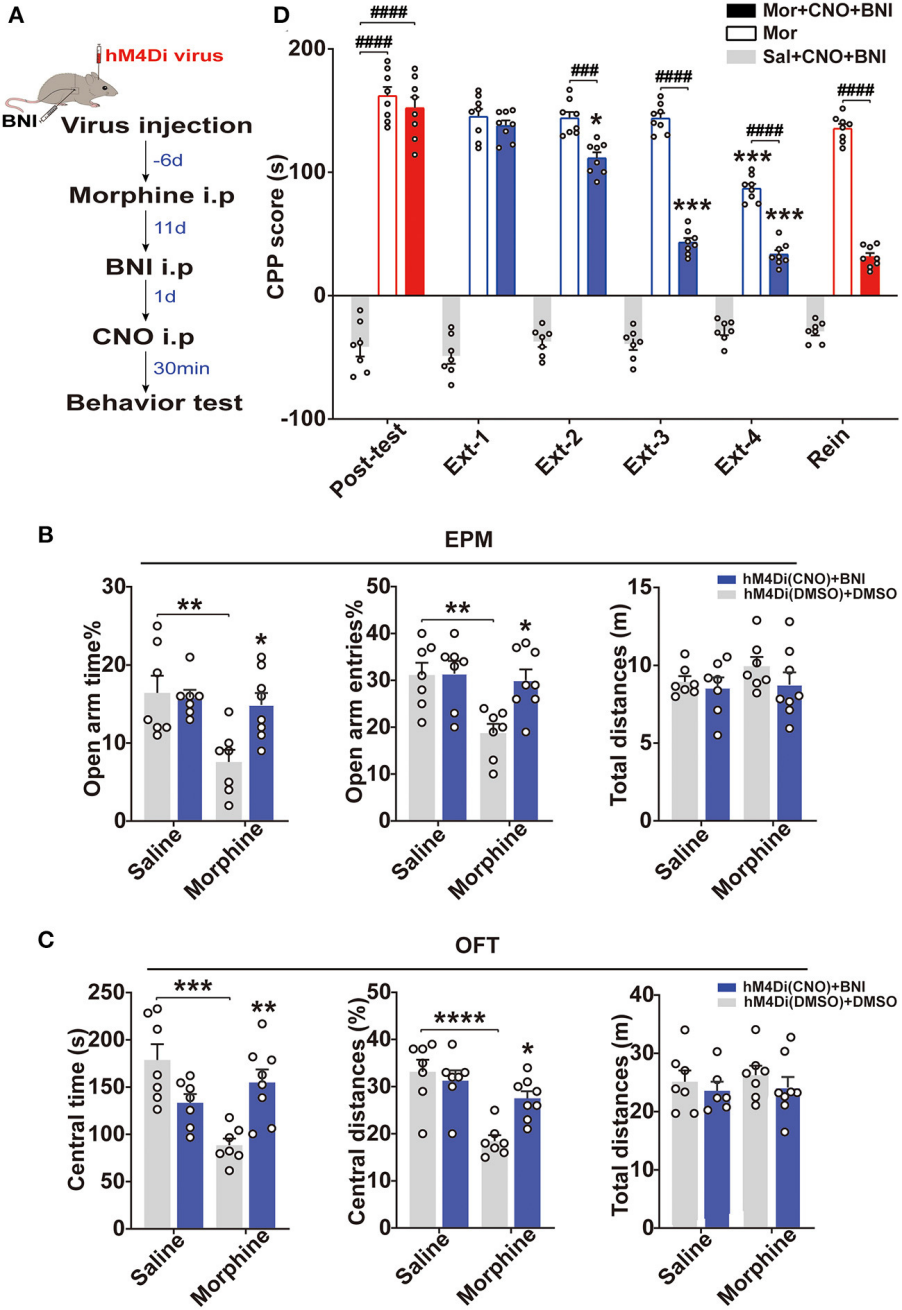
The simultaneous intervention of KORs and the BLA to vHip projections prevented the reinstatement of morphine CPP and anxiety-like behaviors in morphine-withdrawn mice. **(A)** Timeline of experiment. **(B)** The inhibition of the BLA to vHip projections combined with nor-BNI administration increased the open-arm time in and entries into the EPM by the Mor-A mice. **(C)** The inhibition of the BLA to vHip projections combined with nor-BNI administration increased the time spent and distances traveled in the central area of the OFT by the Mor-A mice. In **(B,C)**, the blue histogram represents the hM4Di (CNO) + BNI group, in which the mice were first injected with the chemogenetic virus (hM4Di). Then, the mice morphine withdrawal anxiety model was constructed, and BNI was injected 2 weeks after withdrawal. The next day, the mice were injected with CNO and tested for anxiety-like behavior 30 min later. CNO and BNI were dissolved in 0.5% DMSO. The white histogram represents the hM4Di (DMSO) + DMSO group. **(D)** The inhibition of the BLA to vHip projections combined with nor-BNI administration prevented the reinstatement of morphine CPP. nor-BNI: a long-lasting KOR antagonist. Data are expressed as the mean ± SEM. In the CPP results, *: comparison between the extinction group and the post-test group. #: comparison between the 2 indicated groups. *n* = 7–10/group. *P < 0.05, **P < 0.01, ***P < 0.001, ****P < 0.0001, ^###^*P* < 0.001, ^####^*P* < 0.0001.

Generally, KORs inhibit adenylate cyclase activity by interacting with inhibitory Gα subunits to decrease cell excitability and neurotransmitter release (Crowley and Kash, 2015). Therefore, we hypothesized that the BLA to vHip projections and KORs co-regulated the reinstatement of morphine CPP. To test this hypothesis, we combined chemogenetic approaches with *in vivo* pharmacological manipulations (nor-BNI, a long-lasting KOR antagonist, 10 mg/kg) and assessed anxiety-like behaviors and morphine CPP (Figure 5A). Interestingly, a two-way ANOVA revealed that the combined approach improved anxiety-like behaviors and prevented the stress-induced reinstatement of morphine CPP (*p* < 0.0001, Figures 5B–D). Systemic injection of the nor-BNI alone may prevent the stress-induced morphine CPP reinstatement independent of the BLA to vHip projection, but a synergistic action of the projection and the KORs is possible.

## Discussion

We demonstrated that inhibition of the BLA to vHip projections in mice represented an essential neural substrate for anxiety-like behaviors during morphine withdrawal. KORs in the BLA inputs to the vHip were involved in the anxiety-like behaviors after morphine withdrawal. The morphine-withdrawn mice with obvious anxiety-like behaviors were particularly prone to stress-induced reinstatement of morphine CPP. Furthermore, combined treatment of inhibition of BLA to vHip projections and administration of a KOR antagonist ameliorated anxiety-like behaviors and prevented stress-induced CPP reinstatement after morphine withdrawal.

Anxiety is a critical negative emotional state that emerges during drug withdrawal. In rodents, the EPM is one of the most common protocols used to screen the anxiolytic effects of drugs (Lister, 1987). The EPM is also useful for investigating the biological basis of addiction and withdrawal (Schulteis et al., 1998; Zanos et al., 2016; Masukawa et al., 2020). In our study, anxiety-like behaviors appeared during the first 2 weeks after morphine withdrawal and affected stress-induced reinstatement to drug seeking. This observation indicated that reinstatement could be caused by negative affective states that drive motivated behaviors (Koob and Le Moal, 2008; Koob and Volkow, 2010; Martins et al., 2012). Immunostaining in this study revealed significantly increased numbers of c-Fos-positive cells within the BLA to vHip projections of the Mor-A mice. The BLA is a central component of the neural circuitry governing anxiety-related information and is involved in alcohol (Harper et al., 2019), cocaine (Ladron de Guevara-Miranda et al., 2016), amphetamine (Navarro et al., 2004), and morphine (Niu et al., 2017) withdrawal-related behaviors. One region downstream of the BLA that has been implicated in anxiety-like behaviors in rodents is the vHip (Felix-Ortiz et al., 2013). The vHip contributes to increased anxiety-like behaviors during morphine (Zarrindast et al., 2010) and amphetamine withdrawal (Bray et al., 2016). Although these regions have been associated with addiction and anxiety, there is no previously published evidence demonstrating how these brain areas interact to maintain anxiety-like behaviors during addiction.

*In vivo* optogenetic/chemogenetic inhibition of BLA terminals in the vHip reduced anxiety-like behaviors and accelerated the decreased rate of morphine-paired preference, suggesting that BLA inputs to the vHip were required to maintain basal levels of anxiety-related behaviors after morphine withdrawal. Previous reports revealed that photostimulation of BLA projection neurons targeting the vHip increased anxiety-like behaviors, whereas photosilencing this pathway had the opposite effect (Felix-Ortiz et al., 2013; Namburi et al., 2015). The BLA controls numerous behaviors, including anxiety and reward seeking, *via* the activity of glutamatergic principal neurons. A previous study showed that intra-vHip glutamate receptor antagonism attenuated the effects of optogenetic stimulation, demonstrating that glutamatergic transmission from the BLA to the vHip was critical for mediating light-induced changes in social behaviors (Allsop et al., 2014). These findings demonstrated that excitatory projections from the BLA to the vHip were sufficient to mediate anxiety (Felix-Ortiz et al., 2013). The amygdala also has been central to concepts involving addiction, where it has been proposed to mediate craving and the abnormal attribution of motivational significance to drug-associated cues and contexts (Torregrossa et al., 2011). The hippocampus plays a critical role not only in learning and memory but also in the acquisition and expression of reward-related learning in the process of drug abuse and addiction (Farr et al., 2000; Nestler, 2001b; Moron et al., 2010). We found that inhibition of the BLA to vHip circuit accelerated the decreased rate of morphine-paired preference but did not prevent reinstatement in morphine CPP. Therefore, this study identified that the BLA to vHip circuit governs anxiety-like behaviors after morphine withdrawal.

The KORs in the BLA are an attractive target for neural influence over stress-related behaviors and emotional regulation (Knoll et al., 2011). Evidence has indicated that KORs regulate the neuronal activity of BLA outputs (Knoll et al., 2011), suggesting an important role of KOR within the BLA in opioid withdrawal-related behaviors. Based on the KOR mRNA *in situ* hybridization analysis, the KORs in the BLA afferents onto the vHip were increased in the Mor-A mice. We further found that the knockdown of KORs in the BLA to vHip projections prevented anxiety-like behaviors in the Mor-A mice. Indeed, BLA KOR signaling was necessary and sufficient for KOR-mediated anxiety (Knoll et al., 2011; Tejeda et al., 2015). Furthermore, KOR antagonism in the BLA produced anxiolytic effects (Bruchas et al., 2009; Carroll and Carlezon, 2013). Thus, KORs might mediate negative affective states by modulating neuronal activity in the BLA to vHip projections. We provided functional evidence that KOR knockdown in the BLA synapses in the vHip modulated anxiety-like behaviors in the morphine-withdrawn mice.

The KOR antagonists might have therapeutic benefits for treating mood disorders and drug addiction by promoting stress resilience (Carroll and Carlezon, 2013). However, less is known about the effect on opioid relapse when KOR antagonists are administered in combination with circuit inhibition. Our results indicated that BLA inputs to the vHip were capable of modulating anxiety-like behaviors after morphine withdrawal. Therefore, we used a novel combined approach (inhibition of BLA to vHip projections and KOR antagonism) to prevent stress-induced CPP reinstatement in the Mor-A mice. The results demonstrated that at the system level, projection inhibition (chemogenetic) combined with nor-BNI injections (i.p., 10 mg/kg) ameliorated anxiety-like behaviors and prevented stress-induced reinstatement of morphine CPP. Systemic injection of the nor-BNI alone may prevent the stress-induced morphine CPP reinstatement independent of the BLA to vHip projection, but a synergistic action of the projection and the KORs is possible. Thus, our novel strategy of pathway-specific inhibition with KOR antagonism might prove beneficial in treating anxiety symptoms and relapse during opioid withdrawal.

Collectively, our findings provided a greater understanding of the pathway specificity that underlies emotional valence following chronic morphine cessation and the function of KORs in the generation of negative affective states. The results established a combined protocol that used a chemogenetically inspired approach and KOR antagonist administration that effectively controlled the anxiety-like behavioral phenotype and stress-induced reinstatement of morphine CPP. Our findings suggested novel targets for treating comorbidities of anxiety and opioid addiction.

## Limitations

The BLA sends projections to several limbic, cortical, and thalamic regions, including the ventral hippocampus (vHip) (Felix-Ortiz et al., 2013), the medial prefrontal cortex (mPFC) (Felix-Ortiz et al., 2016), and the lateral hypothalamic area (LHA) (Jimenez et al., 2018). Although the BLA to vHip projections were studied in this study, other BLA projections required additional investigation. In addition, naloxone, an opioid antagonist, was used to induce the morphine somatic withdrawal syndrome (Laschka et al., 1976; Koob et al., 1992; Iyer et al., 2020). Thus, the effect of naloxone on the anxiety-like behavior after morphine withdrawal should also be studied. Furthermore, the specific synaptic plasticity mechanism of the BLA to vHip projections and the molecular mechanism of KORs activation need to be further investigated.

## Data Availability Statement

The original contributions presented in the study are included in the article/Supplementary Materials, further inquiries can be directed to the corresponding authors.

## Ethics Statement

The animal study was reviewed and approved by the Institutional Animal Care and Use Committee of Xi'an Jiaotong University.

## Author Contributions

YZ and JLa designed this study, revised the manuscript, and suggestions for the manuscript. CD, YJ, and XY conducted behavior tests and molecular experiments. CD, YF, and YL conducted chemogenetic and optogenetic experiments. PY, JLi, and SW did the statistical analysis. All the authors reviewed and approved the final version of the publication.

## Funding

This work was supported by grants from the National Natural Science Foundation of China (No. 82001999) and the Natural Science Foundation of Shaanxi Province (Nos. 2020SF-132 and 2020JM-007).

## Conflict of Interest

The authors declare that the research was conducted in the absence of any commercial or financial relationships that could be construed as a potential conflict of interest.

## Publisher's Note

All claims expressed in this article are solely those of the authors and do not necessarily represent those of their affiliated organizations, or those of the publisher, the editors and the reviewers. Any product that may be evaluated in this article, or claim that may be made by its manufacturer, is not guaranteed or endorsed by the publisher.
